# Soil microbial restoration strategies for promoting climate‐ready prairie ecosystems

**DOI:** 10.1002/eap.1858

**Published:** 2019-03-04

**Authors:** Kathryn M. Docherty, Jessica L. M. Gutknecht

**Affiliations:** ^1^ Department of Biological Sciences Western Michigan University 1903 West Michigan Avenue, Mailstop 5410 Kalamazoo Michigan 49008 USA; ^2^ Department of Soil, Water, and Climate University of Minnesota Twin Cities St. Paul Minnesota 55108 USA

**Keywords:** bacteria, climate, fungi, microbe, prairie, restoration, soil, temperature

## Abstract

Tractable practices for soil microbial restoration in tallgrass prairies reclaimed from agriculture are a critical gap in traditional ecological restoration. Long‐term fertilization and tilling permanently alter soil bacterial and fungal communities, requiring microbe‐targeted restoration methods to improve belowground ecosystem services and carbon storage in newly restored prairies. These techniques are particularly important when restoring for climate‐ready ecosystems, adapted to altered temperature regimes. To approach these issues, we conducted a multi‐factorial greenhouse experiment to test the effects of plant species richness, soil amendment and elevated temperature on soil microbial diversity, growth, and function. Treatments consisted of three seedlings of one plant species (*Andropogon gerardii*) or one seedling each of three plant species (*A. gerardii*,* Echinacea pallida*,* Coreopsis lanceolata*). Soil amendments included cellulose addition, inoculation with a microbial community collected from an undisturbed remnant prairie, and a control. We assessed microbial communities using extracellular enzyme assays, Illumina sequencing of the bacterial 16S rRNA gene, predicted bacterial metabolic pathways from sequence data and phospholipid fatty acid analysis (PLFA), which includes both bacterial and fungal lipid abundances. Our results indicate that addition of cellulose selects for slow‐growing bacterial taxa (*Verrucomicrobia*) and fungi at ambient temperature. However, at elevated temperature, selection for slow‐growing bacterial taxa is enhanced, while selection for fungi is lost, indicating temperature sensitivity among fungi. Cellulose addition was a more effective means of altering soil community composition than addition of microbial communities harvested from a remnant prairie. Soil water content was typically higher in the *A. gerardii* treatment alone, regardless of temperature, but at ambient temperature only, predicted metagenomics pathways for bacterial carbon metabolism were more abundant with *A. gerardii*. In summary, these results from a mesocosm test case indicate that adding cellulose to newly restored soil and increasing the abundance of C_4_ grasses, such as *A. gerardii*, can select for microbial communities adapted for slow growth and carbon storage. Further testing is required to determine if these approaches yield the same results in a field‐level experiment.

## Introduction

Grasslands comprise over 25% of the global terrestrial landscape (Singh et al. 1983) and are an important resource for sequestering and retaining terrestrial carbon (Schimel et al. 1994, Amthor and Huston 1998, Jones and Donnelly 2004). In undisturbed grasslands, this functionality is maintained through interactions between the plant and soil microbial communities, including both bacteria and fungi. However, disturbance can alter plant–microbial interactions, leading to permanently altered soil carbon dynamics in grassland systems (Kucharik et al. 2006). These carbon‐rich systems may be further stressed or degraded as climate changes occur, such as increased temperature and altered precipitation regimes (e.g., Rustad et al. 2001, Jones and Donnelly 2004, Zhou et al. 2006, Crowther et al. 2016). Nowhere is this disruption more obvious than in the tallgrass prairie ecosystems that once dominated the Midwestern United States. Beginning in the 1830s, European settlers expanding westward altered much of this ecosystem by suppressing fire (Briggs et al. 2005) and replacing native plants and animals with agricultural crops (e.g., Herkert 1994, Knapp et al. 1999, Chapman and Brewer 2008, Grixti et al. 2009, Koper et al. 2010). This shift from natural to agricultural ecosystems dramatically altered soil microbial communities through long‐term tilling and fertilization (Fierer et al. 2013, Dai et al. 2018, House and Bever 2018), and changed soil physical and chemical parameters resulting in substantial carbon loss to the atmosphere (Post and Kwon 2000). In sum, an estimated 80–99.9% of tallgrass prairies and their accompanying soils have been altered by human activity, making it the most impacted ecosystem in North America (Sampson and Knopf 1994, Sampson et al. 2004).

Efforts to restore tallgrass prairies on reclaimed agricultural land, conservation reserve program (CRP) land, and as prairie strips within active agricultural fields are widespread. They typically focus on supporting native plant diversity, but not on soil microbial diversity or processes (Eviner and Hawkes 2008, Kardol and Wardle 2010). While traditional tallgrass prairie restorations can promote many visible ecosystem services to some degree, such as improved water quality and retention (Vaché et al. 2002, Udawatta et al. 2008) and enriched habitats (Getz and Hofmann 1999, Ries et al. 2001, Fletcher et al. 2002, Vogel et al. 2007, Harmon‐Threatt and Chin 2016), many soil‐based metrics of success, such as soil carbon storage, are not improved with typical restoration strategies (Camill et al. 2004). The lack of high quality soils, which provide the ecosystem service of a net carbon sink, may lead to less resilient systems in the face of climate change (Williams et al. 2016). Best practices for restoring (or reconstructing) soil microbial communities that support desired functions in a changing climate, such as carbon storage, have been less of a management priority, but could be key for restoring robust above‐ and belowground tallgrass prairies.

For most soil biogeochemical and community metrics, restored agricultural soils fall somewhere in between agricultural soils and prairie remnants (McKinley et al. 2005). For example, establishing native plant communities on former cropland improves some key soil microbial properties, such as increasing soil microbial biomass and C mineralization rates, shifting fungal communities and transient increases in C storage (e.g., Baer et al. 2002, Allison et al. 2005, Plassart et al. 2008, Jangid et al. 2010, Morriën et al. 2017). Re‐establishing native prairie plants also promotes soil methanogen populations, leading to greater methane consumption than in active agricultural fields (Werling et al. 2014). However, while initial establishment of early‐successional plants can increase soil carbon storage, restored prairies have higher soil respiration rates and store significantly less soil carbon than remnants (Kucharik et al. 2006) and the rate of carbon accumulation in restored systems decreases over the longer term (Jastrow 1996, Bruce et al. 1999, Post et al. 2004, Kucharik 2007). Across a chronosequence of restored prairies, total soil carbon also did not differ from nearby agricultural fields (Camill et al. 2004), and soil carbon accumulation rates decreased with restoration age, due to turnover of early‐successional plant species (Hernández et al. 2013).

This partial, at best, improvement of soil‐related ecosystem properties after restoration is likely because of long‐term readaptation of microbial communities under agricultural management. Through decades of fertilization, tilling, and agrochemical use, soil microbial communities in agricultural systems have become so altered that they are thought to reach an alternative stable state (Jangid et al. 2010, Fichtner et al. 2014). For example, nitrogen fertilization causes a consistent decrease in soil bacterial diversity and microbial biomass carbon, independent of nitrogen application rates or crop types (Dai et al. 2018). Nitrogen fertilization is also related to increased relative abundances of *Proteobacteria* and *Actinobacteria* (Ramirez et al. 2010, Zhou et al. 2017, Dai et al. 2018), which include taxa that are adapted to high nitrogen demand and labile carbon pool metabolism (Fierer et al. 2007, Collins et al. 2016). Conversely, in North American remnant prairies, where plant organic material inputs control the growth of these copiotrophic taxa, *Verrucomicrobia*, which are thought to specialize in recalcitrant carbon degradation, are more predominant members of the community (Fierer et al. 2013). Mechanical disruption of soils through tilling also impacts soil arbuscular mycorrhizal fungi (AMF), which symbiotically colonize plant roots, promoting growth of many prairie plants and playing a large role in determining plant community diversity (Vogelsang et al. 2006). Fungal, and especially mycorrhizal, biomass and associated decomposition activity, are among the most significant contributors to soil C storage (Treseder 2016). In remnant prairies that were never disturbed by tilling, soil AMF communities differ significantly across a broad precipitation gradient, indicating that locally adapted AMF communities play an important role in establishing and maintaining the plant community (House and Bever 2018). However, in mechanically disturbed soils, AMF communities are relatively homogeneous across this gradient, possibly leaving these ecosystems more vulnerable to future disturbances, such as climatic changes to the precipitation regime (House and Bever 2018). Taken together, these results suggest that belowground restoration efforts, focusing on promoting soil microbial communities adapted for carbon storage, are required.

Climate change presents an increased urgency to the restoration of belowground ecosystem services such as carbon storage and soil quality. As both natural and disturbed grassland ecosystems face the effects of climate changes, such as warming, soil respiration rates will likely increase (Bond‐Lamberty and Thomson 2010, Luo et al. 2014), with the consequence of reducing soil carbon stores, depending on temperature sensitivity (Carey et al. 2016). This is because soil microorganisms exhibit changes in physiological properties when they are warmed, including less resource allocation to biomass and degradative enzyme production (Allison et al. 2010). These physiological changes are counteracted by shifts in carbon‐use efficiency and changes in community structure (e.g., Zogg et al. 1997, Allison et al. 2010), which leads to the observed reduction in temperature sensitivity over time (e.g., Luo et al. 2001). Above a certain temperature threshold, heat stress can also limit the observable change in temperature sensitivity (Schimel et al. 2007, Balser et al. 2010). Because agricultural systems alter microbial carbon cycling in dramatic and long‐lasting ways, even after restoration to native plant communities, measurable temperature sensitivity may also be altered in restored systems that were previously under agricultural management.

Microbially conscious restoration can help land managers reconnect above‐ and belowground dynamics, possibly creating ecosystems that are more resilient to climate warming. Recalcitrant carbon addition to the soil is one “prebiotic” strategy that can reduce nitrogen mineralization rates (e.g., Averett et al. 2004), and possibly select for microbial communities with higher abundances of slow‐growing specialist taxonomic groups. A “probiotic” approach, such as adding specific mycorrhizal species to new restorations, stimulates growth of late‐successional prairie plant species, but only promotes initial increases in soil carbon storage in a new restoration (Koziol and Bever 2016, 2017). However, little is known about whether addition of whole communities, including bacteria and fungi, can promote longer‐term carbon storage. To address these questions, we conducted a multi‐factorial greenhouse experiment as a pilot study to examine the effects of plant species richness, soil amendment, and elevated temperature on newly restored soil bacterial and fungal community structure, function, and soil carbon storage. We hypothesized that (1) higher plant‐species richness promotes more diverse soil microbial communities; (2) inoculation with remnant soil microbial communities increases microbial diversity and improves metrics related to carbon storage; (3) amendment with cellulose, a recalcitrant substrate, shifts microbial communities toward greater fungal biomass and bacterial taxa that store carbon more efficiently; (4) higher plant‐species richness, inoculation, and cellulose addition will all recreate soil microbial communities that resist the effects of warming more than low diversity plantings or unamended soils.

## Methods

### Experimental design

In May 2015, we obtained soil from the top 10 cm of an agricultural field that was undergoing a traditional restoration located at the Edward Lowe Foundation in Cassopolis, Michigan, USA. Following collection, we passed all soil through a 2‐mm sieve into large decontaminated plastic containers and then homogenized the soil. We added 10 kg of soil to 72 two‐gallon plastic pots (1 gallon = 3.79 L), and each pot was given a unique ID. We used a multi‐factorial approach to test for the effects of the number of plant species (two levels), temperature (two levels) and soil amendment (three levels) on abiotic and biotic soil parameters. Each soil amendment treatment was fully crossed with each plant species and temperature treatment. We prepared six replicate pots for each treatment, for a total number of 72 pots. All treatments were prepared within 2 weeks of soil collection.

### Plant species treatments

We used three native tallgrass prairie plants to test for the effect of plant species richness on restored prairie soils: *Andropogon gerardii* (Big bluestem grass), *Echinacea pallida* (purple coneflower), and *Coreopsis lanceolata* (lance‐leaved coreopsis). Germinated seedlings of each species, grown in fertilized potting soil, were purchased from Cardno Native Plant Nursery (Walkerton, Indiana, USA). Prior to transplanting into the experimental pots, soil was removed from the seedling roots by hand, they were rinsed first in water and then in a 10% detergent solution, and the mass of each seedling was recorded. We planted seedlings into one of two treatments: one plant species, which consisted of three *A. gerardii* seedlings, and three plant species, which consisted of one *A. gerardii*, one *E. pallida* and one *C. lanceolata* seedling. The potted plants were kept at ambient temperature in Finch Greenhouse (Western Michigan University, Kalamazoo, MI). The plants were grown for one week prior to beginning temperature manipulation and soil amendments. We maintained field levels of soil moisture (10%) by adding 200 mL of reverse osmosis (RO) water to each pot every other day.

### Soil amendment treatments

We prepared three types of soil amendments: (1) addition of live remnant prairie microorganisms (live inoculation), (2) addition of autoclave‐killed remnant prairie microorganisms (inoculation control), and (3) addition of cellulose. (We also prepared a second control, which only had 1× PBS added, but no variables differed between the inoculation control and the PBS control, so only the results of the inoculation control are presented here.) To prepare the remnant‐soil microorganism inoculates, we collected soil from a remnant prairie that has never been used for agricultural purposes, located in Kalamazoo, Michigan. We collected 5 kg of soil from the top 10 cm and passed soil through a 2 cm sieve. All soils were collected in late May, at the same time that the agricultural soils were collected. Remnant soil was stored in a sterile container in the dark at ambient temperature for <2 weeks until experimental set‐up could be completed. Sterile water was added to keep the remnant soil at 10% soil moisture. We prepared both the live inoculate and the autoclave‐killed control inoculate on the same day, and also froze remnant soil for community analyses. To test the effects of microbial addition alone without adding remnant soil, which contains abiotic nutrients and organic matter, we conducted a cellular extraction procedure to prepare our treatments. First, we conducted an extraction to displace nutrients from the remnant soil: we added 100 g of sieved remnant soil to each of 48 1‐L sterile Pyrex bottles, and added 500 mL of 0.5 mol/L CaCl_2_ to each bottle. These were placed on a shaker table for 1 h at 100 rpm at room temperature. Following extraction, we allowed the bottles to settle for 5 min to separate the aqueous layer from the solid soil and decanted the extractant, leaving 50 mL of extractant in the bottle. Then we conducted a second extraction to isolate live cells from the soil. We added 250 mL of sterile 1× PBS buffer and four 2 cm diameter sterilized glass beads to each of the 1‐L bottles + nutrient‐extracted soil. We placed the bottles on a shaker table again for 1 h at 100 rpm to conduct the cellular extract. Following extraction, we allowed the bottles to settle and then decanted off the supernatant from 24 of the 48 bottles into a single acid‐washed and decontaminated plastic carboy. To prepare the final live soil inoculate (treatment 1), we homogenized the supernatant by shaking it at 100 rpm for 1 h. In the meantime, to prepare the autoclave‐killed control soil inoculate (treatment 2), we decanted the supernatant from the remaining 24 1‐L bottles into a single glass flask and autoclaved it for 30 min to kill the live cellular extract. After it cooled, we homogenized the autoclaved supernatant by shaking it at 100 rpm for 1 h. The autoclaved killed control was used to determine whether nutrients or some other soil property was responsible for treatment effects instead of the microbes themselves.

The cellulose addition treatment served to ask the question of whether the addition of a complex carbon source could act to recondition or to reactivate the microbial communities already present in agricultural soils. To prepare the cellulose amendment (treatment 3), we suspended 1 kg of cellulose microcrystalline (Acros Organics 9004‐34‐6; Morris, NJ, USA) into 5 L of sterile 1× PBS in a sterilized plastic carboy. We shook the carboy for 1 h at 100 rpm to ensure that the cellulose was dissolved. Once all soil amendment treatments were prepared, we manually aerated the potted soil and added 200 mL of each different solution to 12 replicate pots for each of the two plant treatments. We note that all amendments included a low‐level addition of phosphorus contained in PBS buffer to the soils. However, background soil phosphorus concentrations in all treatments were within the normal range of variation for other restored grasslands in the region (20–200 μg TP/g dry soil; K.M. Docherty, *unpublished data*).

### Temperature treatments

Once the plant and soil amendments were implemented, we moved six of the replicates for each plant × soil amendment treatment to a different room in the greenhouse. We used a random number generator to determine the placement of pots by their unique IDs. One room was kept at ambient temperature while we elevated the temperature in the other room to + 4°C above ambient. We monitored the temperature and humidity in both rooms throughout the experiment.

### Experiment maintenance and sample collection

We allowed the experiment to run for 62 d prior to experimental analysis. The photoperiod during the time of the experiment corresponded to ambient day length, which was 15 h, 46 min at maximum. Average (± SD) midday air temperature in the ambient room was 29.02° ± 2.47°C and in the warmed room was 31.95° ± 3.38°C. During this time, we added reverse‐osmosis water to all pots to maintain approximately 10% soil moisture; pots in the elevated temperature room were watered more frequently than at ambient temperature to prevent a drought effect. At the end of the experimental period, we aseptically removed 100 g of soil from each pot using a small soil corer, and placed it in a plastic press‐seal bag. The soil sample was homogenized and divided so that 50‐g subsets of fresh soil were used for soil physiochemical and extracellular enzyme activity analyses, 30‐g subsets were dried and used for lipid analysis, and 20‐g subsets of this soil was frozen at −80°C for DNA extraction and 16S rRNA community analysis. On the same day that soil samples were collected, we removed whole plants and roots by hand from each pot. We then washed the roots thoroughly in water to remove soil particles and separated aboveground (AGB) and belowground (BGB) plant material. We dried the two biomass samples in paper bags in the greenhouse for 2 weeks. Following drying, we weighed the mass of AGB and BGB for each plant and calculated root‐to‐shoot ratios. All plant and soil samples were collected over the course of two weeks.

### Soil physiochemical properties

Using fresh soil, we measured soil pH (5 g soil, 20 mL deionized water) with a Dual Channel AR25 pH/ion meter (Fisher Scientific, Waltham, MA, USA). We determined percent soil water content by drying 25 g of fresh soil in aluminum pans at 55°C for 2 weeks, and calculating the mass loss through drying. We measured the percent soil organic matter content by mass loss through combustion at 360°C for 2 h using a muffle furnace (Fisher Isotemp, Waltham, MA, USA). We determined extractable phosphorus content using 2.5 g of fresh soil and 40 mL of 0.5 mol/L NaHCO_3._ Extraction tubes were placed onto a platform shaker for 18 h at 150 rpm, then centrifuged for 5 min at 2326 *g*. We used vacuum filtration and a GF/F (Whatman, GE Healthcare Life Sciences, Kent, UK) filter to collect the filtrate and stored it in a 20‐mL plastic bottle at −20°C prior to analysis. Filtered extracts were analyzed by the UMN Research Analysis Laboratory using molybdate‐blue colorimetric analysis with a Brinkmann PC 900D probe colorimeter at 900 nm wavelength (Frank et al. 1998). Total soil %C, total soil %N, and the subsequent C:N ratio were determined on oven‐dried, ground soil samples using combustion analysis (Elementar pyrocube; Elementar Americas, Ronkonkoma, NY, USA).

### Extracellular enzymatic activities

We assessed the activity of four hydrolytic enzymes using fluorescent‐linked substrates, based on previously described techniques (Sinsabaugh et al. 2005, Gutknecht et al. 2010, German et al. 2011). We tested for activities of cellobiohydrolase using 4‐MUB‐cellobioside, β‐glucosidase using 4‐MUB‐β‐glucopyranoside, N‐acetylglucosaminidase using 4‐MUB‐N‐acetyl‐β‐glucosaminide and phosphatase using 4‐MUB‐phosphate. All substrates were purchased from Sigma‐Aldrich (St. Louis, Missouri, USA). Briefly, we added 1 g of soil to 125 mL of 50 mmol/L Tris buffer (adjusted to pH 7) in a 100 mL centrifuge bottle. We added a stir bar to the bottle and shook the mixture for 1 h at 5,000 rpm. We then added the soil slurry to black 96‐well plates containing 50 mL of 200 mmol/L 4‐MUB‐linked enzyme substrates. We included eight replicate wells for blanks (buffer alone), negative controls (only substrate solution or soil slurry with buffer), quench standards (4‐MUB + soil slurry), and reference standards (4‐MUB with buffer), as described in Gutknecht et al. (2010). After 1 h of reaction time in the dark at 23°C, we added 10 mL of 0.5 mol/L NaOH. We measured fluorescence using a microplate fluorometer (Cary Eclipse) with 260 nm excitation and 465 emission filters. We calculated extracellular enzyme activity as the (nmol substrate cleaved)·(g dry soil equivalent)^−1^·h^−1^.

### Phospholipid fatty acid (PLFA) analysis

Soil microbial biomass and the abundances of broad indicator groups were determined using a modified PLFA FAME protocol (Oates et al. 2017). Lipid analysis was performed for a randomly chosen four out of the experimental six replicates for each treatment. For each sample, 3 g of freeze‐dried soil was used for PLFA extractions. Phospholipids were extracted from the soil three times using a chloroform–methanol–citrate buffer mixture (2:4:1.8 v/v/v), and were then saponified. Strong acid methanolysis (325 mL HCl and 50 mL methanol) was then performed to convert phospholipids into fatty acid methyl esters. Next, we extracted fatty acid methyl esters from the aqueous to the organic phase of the solution using hexane. We used a base wash (300 mmol/L sodium hydroxide solution) to remove impurities. Gas chromatography (CG/MS) was used to identify and quantify fatty acids from each extraction. We used a Zebron ZB‐5 (30 m × 0.32 mm × 0.1 μm) and an Agilent 7890 gas chromatograph coupled to an Isoprime 100 mass‐selective detector (Americas, Mt. Laurel, New Jersey, USA). The C13:0 fatty acid was used as an internal standard to quantify individual peaks. Several lipids can be used as biomarkers for broad microbial groups including fungi (18: 1 ω 9c, 18:2 ω 6,9, 16:1 ω 5c (AMF)), Gram‐negative bacteria (17:0 cyclo, 18:1 ω 9t, 19:0 cyclo, 16:1 ω 9c, 16:1 ω 7c, 16:1 ω 5c), Gram‐positive bacteria (17:0 iso, 17:0 anteiso, 15:0 iso, 15:0 anteiso) and Actinobacteria (16:0 10me) (Zelles 1999). Total microbial biomass was calculated as the sum of all lipids <20 carbons in length.

### DNA extraction, 16S rRNA sequencing and predicted metagenomics

On the day of DNA extraction, we defrosted frozen soils collected from each of the 72 treated pots and extracted total genomic DNA using a PowerSoil DNA Isolation Kit (MoBio, Carlsbad, California, USA) using 0.25 g soil. Randomized samples were extracted on four different days using the same kit, and two negative extraction controls (no soil added) were extracted on each of the four dates. We also extracted DNA from three replicate samples of remnant soil used to prepare the inoculate treatment and autoclave‐killed inoculate control as well as three replicate samples of the agricultural soil prior to experimental manipulations so that we could compare the two communities. We quantified DNA using a Qubit 2.0 fluorometer (Life Technologies, Carlsbad, California, USA) with a dsDNA HS Assay kit (Life Technologies Q32854). Amplicon preparation and MiSeq (Illumina, San Diego, California, USA) sequencing was conducted at Michigan State University Genomics Core Facility. The V4 hypervariable region of the 16S rRNA gene in Bacteria was amplified using primers 515F/806R (Kozich et al. 2013). A subset of PCR products was analyzed on a 1% agarose gel stained with ethidium bromide to ensure that samples contained sufficient DNA for amplification procedures. DNA libraries were normalized using the SequalPrep Normalization Plate Kit, 96‐well (Thermo Fisher Scientific, Waltham, Massachusetts, USA), and samples from each replicate plate were pooled into single wells. Pooled samples were quantified using a Kapa Biosystems qPCR kit (Kapa Biosystems, Wilmington, Massachusetts, USA), and samples were normalized to an equal concentration. Each sample pool was loaded on an Illumina MiSeq flow cell v2 and sequenced using a 500 cycle (PE250) reagent kit. Bases were called using Real Time Analysis (RTA) software v1.18.54, and RTA output was demultiplexed and converted to fastq files using Illumina Bc12Fastq v1.8.4.

Once the raw sequence data was returned to us, we conducted primer sequence removal, quality filtering, and merged forward and reverse reads using PANDAseq version 2.8 (Masella et al. 2012). We excluded sequences from analysis if they contained ambiguous base calls, more than eight identical bases in a row, quality scores of <0.97 in a sliding scale of 0 to 1, fewer than 275 bases, more than 275 bases, or sequence overlap of <47 bases. Fasta files are deposited in the NCBI Sequence Read Archive under accession number PRJNA454440, and are publicly available. We identified and filtered chimeric sequences with QIIME v.1.9.1‐2 (Caporaso et al. 2010) using the vsearch algorithm (Rognes et al. 2016). We clustered the remaining sequences into operational taxonomic units (OTUs) using the pick_open_reference_otus.py script in QIIME, which selected open‐reference OTUs and assigned taxonomic information to OTUs using the Ribosomal Database Project classifier (Wang et al. 2007) against the SILVA 128 reference database using a 97% cutoff for OTU classification (Quast et al. 2013). We removed all sequences associated with the eight extraction negative control samples from analysis, as well as any sequences identified as Archaea, chloroplasts, mitochondria, or within the genus *Ralstonia*, which are common contaminants in DNA extraction kits (Salter et al. 2014). Following this clean‐up of the data set, the number of sequences per sample ranged from 10,719 to 86,393.

To prepare the data for analysis of predicted metagenomics pathways, we also conducted the same steps as described above, except that closed‐reference OTUs were selected and taxonomic information was assigned using the Greengenes version 13.8 reference database (DeSantis et al. 2006). We used the resulting OTU table to predict potential metagenomics pathways (KEGG 1‐3) using the Phylogenetic Investigation of Communities by Reconstruction of Unobserved States (PICRUSt) tool, version 1.1.3 (Langille et al. 2013).

### Data analysis and statistics

All statistical analyses were conducted using the R statistical environment (version 3.4.3). Our experimental design contained six replicates for each plant × soil amendment treatment; our temperature treatments were implemented by room and were not replicated. Therefore, for univariate measurements, we split our analyses so that significant differences among the plant × soil amendment treatments were assessed at each temperature using ANOVA and Tukey's post hoc tests. When appropriate, we tested for the effect of temperature and interactions with temperature using a generalized linear model (GLM) approach. All multivariate statistics were conducted using the vegan package, version 2.4‐6 (Oksanen et al. 2018) and we visualized all multivariate data using principal coordinates analyses (PCoA). For PLFA, we calculated the relative abundances of lipids within each sample and used the Bray‐Curtis dissimilarity approach (e.g., Faith et al. 1987). We then used permutational analysis of variance (PERMANOVA) to determine significant differences between each of the plant × soil amendment treatments within each temperature treatment, and to test for interactions. We also examined whether PLFA‐based communities differed by temperature. For 16S rRNA community analysis, we calculated a weighted UniFrac distance matrix (Lozupone and Knight 2005) using QIIME, and used the phyloseq (version 1.16.2) package to run PERMANOVAs to determine significant differences in β‐diversity between the plant × soil amendment treatments within each temperature treatment and to test for interactions. When significant, we used pairwise PERMANOVA tests to examine the underlying significant differences in α‐diversity, using the RVAideMemoire (version 3.4.4) package (Hervé 2018). We calculated OTU richness and Faith's phylogenetic diversity using picante, version 1.6‐2 (Kembel et al. 2010). Finally, we identified all Kyoto Encyclopedia of Genes and Genomes (KEGG1) pathways related to metabolism, as predicted by the 16S rRNA data set (Kanehisa et al. 2016). We calculated the relative abundances of each metabolic pathway at the KEGG3 level and used PERMANOVA to determine whether there were differences between plant × soil amendment treatments at each temperature, and to test for interactions. When significant, we used ANOVA to determine which pathways differed by plant treatment or by soil amendment treatment. We also examined whether predicted pathways differed by temperature.

## Results

Remnant soil bacterial communities differed significantly and were more diverse than restored soil communities (Appendix S1: Fig. S1A). In the soils we examined, relative abundances of *Bacteroidetes*,* Betaproteobacteria*,* Verrucomicrobia*, and *Planctomycetes* were higher in the remnant soil used to prepare the inoculate. Relative abundances of *Acidobacteria*,* Firmicutes*,* Chloroflexi*, and *Gemmatimonadetes* were higher in the agricultural soil (Appendix S1: Fig. S1B). Copiotrophic phyla adapted for rapid growth and higher potential for labile carbon metabolism in high nutrient conditions are *Betaproteobacteria*,* Bacteroidetes*,* Firmicutes*,* and Actinobacteria*. Oligotrophic phyla adapted for slow growth and higher potential for carbon storage are *Acidobacteria* and *Verrucomicrobia* (Fierer et al. 2007, Collins et al. 2016). Other phyla cannot be categorized into either group.

### Treatment effects at ambient temperature

#### Plant species

**Figure 1:**
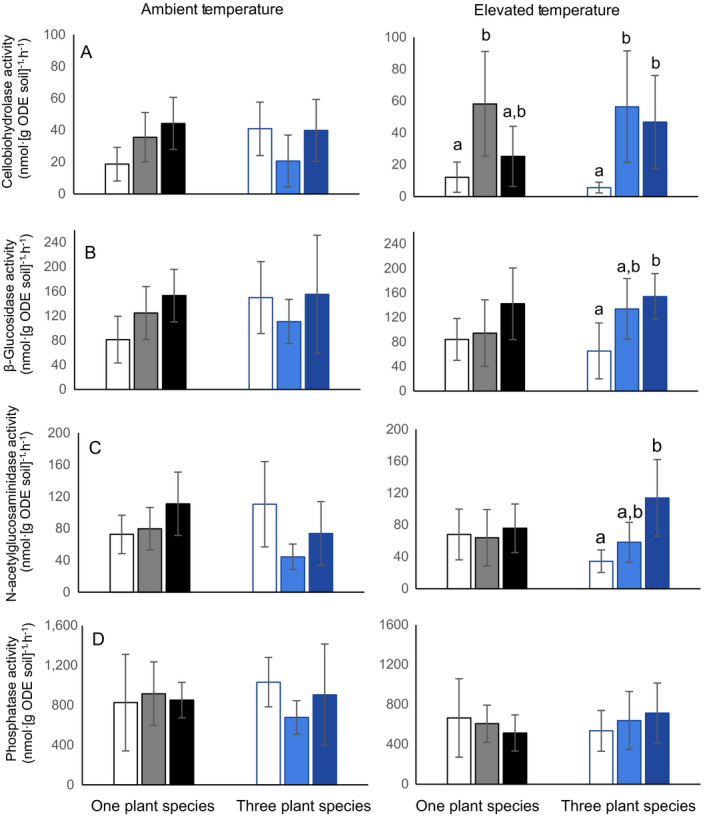
Extracellular enzyme activities (EEA) for four hydrolytic enzymes for all experimental treatments, expressed as the average activity ± 95% confidence intervals. Treatments are control (white), remnant inoculate (hatching), and cellulose (black), per plant treatment (1‐plant species is black; 3‐plant species is blue), and by ambient (left) and elevated (right) temperatures. Significance differences at α = 0.05 are indicated by different lowercase letters above bars. At ambient temperature, there is no effect of plant treatment or soil amendment on any of the EEAs measured. At elevated temperature remnant inoculation and cellulose amendment correspond with increases in (A) cellobiohydrolase activity, particularly when three plant species are present (*P* < 0.009). (B) β‐glucosidase and (C) N‐acetylglucosaminidase activities are highest when cellulose is added in the three‐plant‐species treatment only at elevated temperature (*P* < 0.03). (D) There is no effect of any treatment on phosphatase activity (*P* > 0.783). Data S1.

**Figure 2:**
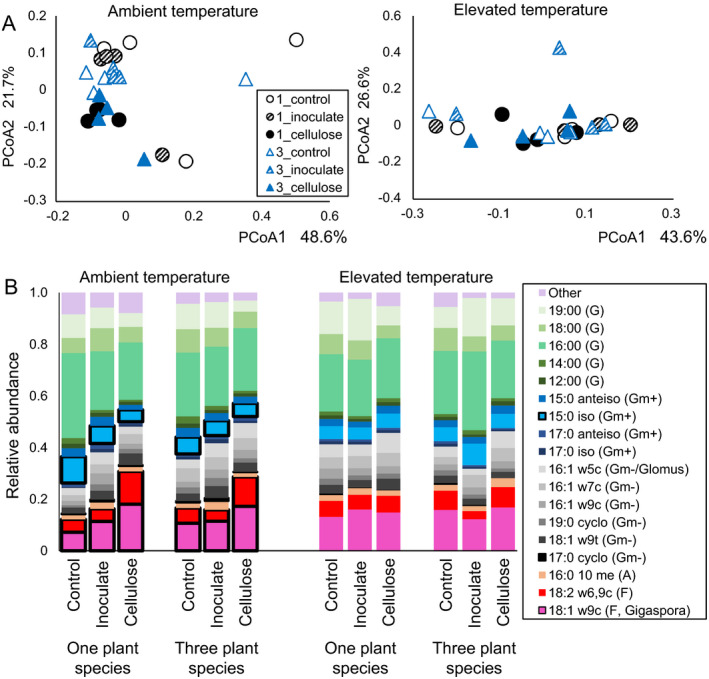
(A) Principal coordinates analysis ordinations of lipid‐based microbial communities for all crossed plant (one plant species is black; three plant species is blue) × soil amendment treatments at ambient (left) and elevated (right) temperatures. Lipids are grouped by fungal (F), Actinobacterial (A), Gram‐negative bacterial (Gm‐), Gram‐positive bacterial (Gm+), general microbial (G) and other lipids. At ambient temperature, there is a significant effect of the soil amendment treatment only (PERMANOVA, *P* = 0.012), but at elevated temperature, there is no effect (*P* = 0.414). Plant treatments did not affect overall community composition. (B) Proportional relative abundance of the fungal and bacterial lipids identified using phospholipid fatty acid analysis (PLFA). At ambient temperature only, there are significant effects of the cellulose treatment (indicated with heavy black lines) on 15:0 iso (decrease, *P* = 0.006), 17:0 cyclo (decrease, *P* = 0.025), 18:1 ω9c (increase, *P* < 0.0009), and 18:2 ω6,9c (increase, *P* < 0.003). 16:1 ω7c is more abundant in the three‐plant‐species treatment than in the one‐plant‐species treatment at ambient temperature (*P* = 0.04). Data S2.

**Figure 3:**
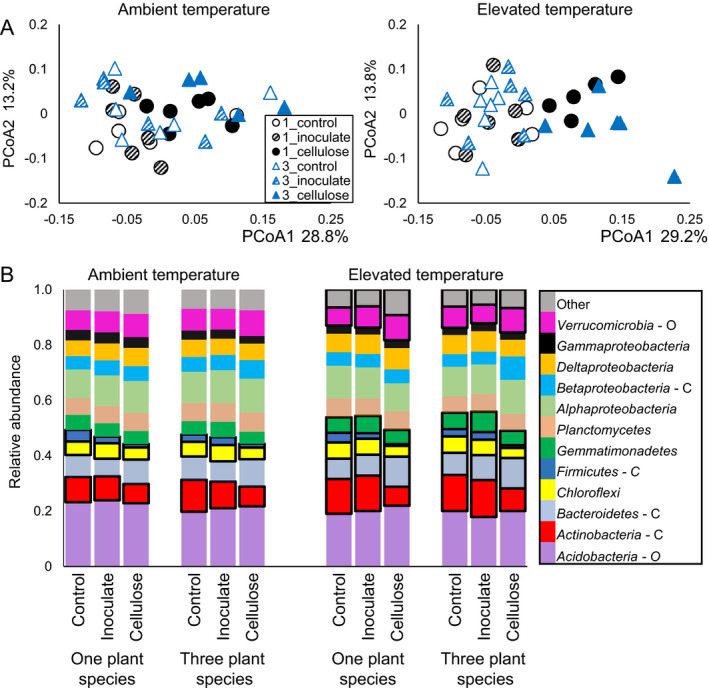
(A) Principal coordinates analysis ordinations based on weighted UniFrac distances between bacterial communities identified using 16S rRNA amplicon sequencing for all crossed plant (one plant species is black; three plant species is blue) × soil amendment treatments at ambient (left) and elevated (right) temperatures. Significance is assessed at α = 0.05. At ambient temperature, there is a significant effect of soil amendment (*P* = 0.003) and an interaction between the plant and soil amendment treatments (*P* = 0.024), but not an independent effect of the number of plant species (*P* = 0.106). The same pattern occurs at elevated temperature, with more significant effects of soil amendment (*P* = 0.001) and the plant × soil amendment interaction (*P* = 0.001). (B) Proportion relative abundance of bacterial phyla that comprise >5% total abundance in all treatments. Phyla that differ significantly across soil amendment treatments are indicated with bold lines. Phyla indicated with O in the legend are classified as oligotrophic; those indicated with C are classified as copiotrophic.

**Figure 4:**
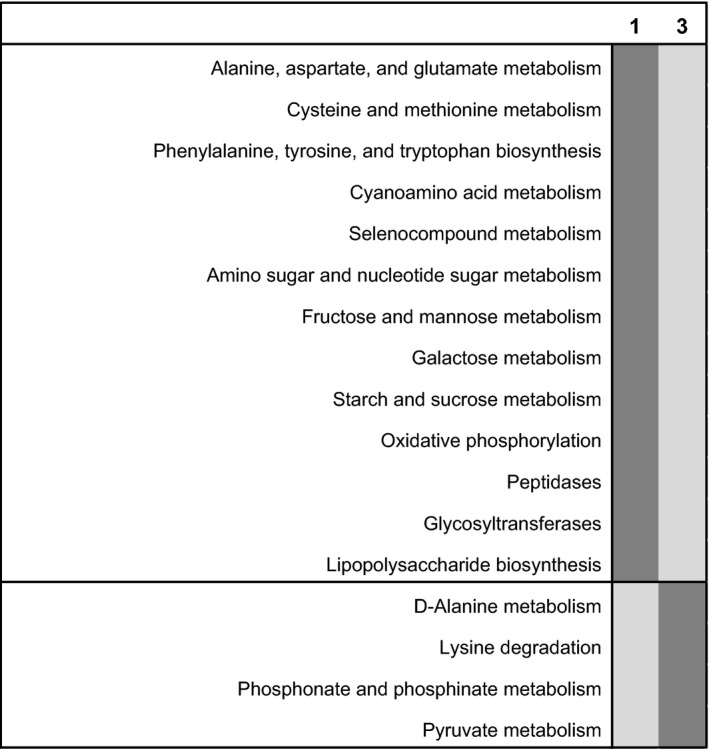
Heatmap indicating significant differences in the proportion of metabolic pathways predicted from 16S rRNA sequence data for the two plant‐species treatments (one species [1] vs. three species [3]) at ambient temperature. Significance is assessed at α = 0.001, dark shading indicates that the proportion of the predicted pathway is higher. Data S3.

The number of plant species did not change total AGB; in the three‐plant‐species treatment, shoot biomass of *C. lanceolata* was higher than the shoot biomass of the other two plant species (Appendix S1: Fig. S2). Total BGB was higher in treatment containing only *A. gerardii*, and lower in the three‐plant‐species treatment, but individual seedlings did not differ (Appendix S1: Fig. S3). The only soil physiochemical measurement that differed between the two plant treatments was soil water content; on average, it was 2% higher in the treatments with *A. gerardii* only (Appendix S1: Fig. S4). Plant‐species treatment had no effect on extracellular enzyme activities (Fig. 1), total lipid biomass, fungal : bacterial ratio of lipids in soils (Appendix S1: Fig. S5), or overall lipid‐based community composition (Fig. 2A). The only lipid that differed between the two plant treatments was 16:1 ω7c (Fig. 2B). This indicator for Gram‐negative bacteria was in greater abundance with three plant species than with one plant species (*P* = 0.04). On average, all 16S rRNA‐based bacterial communities were composed of *Acidobacteria* (21%), *Alphaproteobacteria* (11%), *Actinobacteria* (10%), *Bacteroidetes* (9%), *Verrucomicrobia* (8%), *Deltaproteobacteria* (7%), *Planctomycetes* (6%), *Betaproteobacteria* (5%), *Gemmatimonadetes* (5%), *Chloroflexi* (5%), *Gammaproteobacteria* (3%), and *Firmicutes* (2%). OTUs belonging to other phyla or were unassigned comprised an average of 7% of the soil community (Fig. 3). There was no effect of plant treatment on OTU richness (*P* = 0.92). There was only a marginal effect of plant species on 16S rRNA‐based bacterial community structure (*P* = 0.106, Fig. 3A), but the relative abundances of two bacterial phyla differed by plant treatment. *Acidobacteria*, which are classified as oligotrophic, were significantly less abundant in the three‐plant‐species treatment (20.8%) than in the one‐plant‐species treatment (23.3%, *P* = 0.004); relative abundances of *Gammaproteobacteria* were also significantly lower in the three‐plant‐species treatment (3.1%) than in the one‐plant‐species treatment (3.9%, *P* = 0.03, Fig. 3B). Predicted metabolic pathways also differed based on the plant treatment (*P* = 0.007, Appendix S1: Fig. S6). Pathways indicative of amino acid, amino sugar and simple sugar metabolism (fructose, mannose, galactose, sucrose) as well as oxidative phosphorylation, peptidase, and lipopolysaccharide production were significantly higher in the treatment with *A. gerardii* alone (Fig. 4). Pathways for D‐alanine metabolism, lysine degradation, phosphonate/phosphonate metabolism, and pyruvate metabolism were higher in the three‐plant‐species treatment.

#### Soil amendments

Soil amendment treatments at ambient temperature did not have an effect on total AGB or BGB and did not influence the root or shoot biomass of individual seedlings (Appendix S1: Figs. S2, S3). Addition of remnant soil extract did not have an effect that differed from the control for any parameter we measured at ambient temperature. Cellulose addition resulted in higher pH and total phosphorus in both plant treatments and higher SOM in the one‐plant‐species treatment only (Appendix S1: Fig. S4). Cellulose did not have an effect on extracellular enzyme activities (Fig. 1) and did not change the total lipid biomass. However, fungal : bacterial lipid ratios were significantly higher with cellulose addition, regardless of the plant treatment (Appendix S1: Fig. S5). This corresponded with a significant effect of cellulose on lipid‐based community composition (Fig. 2A). Lipids 18:2 ω6,9c and 18:1 ω9c, which are fungal indicators, were higher in cellulose‐amended soils than in the control, while 15:0 iso and 17:0 cyclo, which are bacterial indicators, were lower (Fig. 2B). The 16S rRNA‐based bacterial communities also changed with cellulose addition, as compared to the control (Fig. 3). Relative abundances of *Actinobacteria* and *Firmicutes* (both copiotrophic phyla) and *Chloroflexi* were all significantly lower in the cellulose‐amended soils than in the control (Fig. 3B). The relative abundance of *Verrucomicrobia* (an oligotrophic phylum) was higher in cellulose‐amended soils, but the increase was not significant. Predicted metabolic pathways did not differ with soil amendments at ambient temperature (Appendix S1: Fig. S6).

### Treatment effects at elevated temperature

#### Plant species

Increased temperature altered several plant and microbial factors. Under warmed conditions, plant AGB and BGB were both significantly higher in the treatment with *A. gerardii* alone than in the three‐plant‐species treatments (Appendix S1: Figs. S2, S3). In the three‐plant‐species treatment, shoot biomass *A. gerardii* and *C. lanceolata* were both higher than shoot biomass of *E. pallida* (Appendix S1: Fig. S2) and root biomass of *A. gerardii* was higher than the other two species (Appendix S1: Fig. S3). As at ambient temperature, soil water content was higher in the treatment with *A. gerardii* alone than with three plant species (Appendix S1: Fig. S4), but plant treatment alone did not influence any other soil physiochemical parameters. Extracellular enzyme activities were not influenced by the number of plant species (Fig. 1). Overall, increasing the temperature did not alter lipid‐based community composition, as compared to communities at ambient temperature (Appendix S1: Fig. S7). There was also no significant effect of plant treatment on lipid‐based communities under elevated temperatures (Fig. 2). Total lipid biomass was higher with *A. gerardii* alone than with three plant species, but this did not influence the fungal : bacterial ratio in the soils at elevated temperature (Appendix S1: Fig. S5). Though 16S rRNA‐based communities differed under ambient and elevated temperatures (Appendix S1: Fig. S8), there was no significant effect of plant species on OTU richness (*P* = 0.907) or 16S rRNA‐based bacterial community structure (*P* = 0.271). As at ambient temperature, there was an interaction between plant and soil amendment treatments (*P* = 0.001, Fig. 3). In contrast to the results at ambient temperature, there was no effect of plant treatment on predicted metabolic pathways (Appendix S1: Fig. S6).

#### Soil amendments

**Figure 5:**
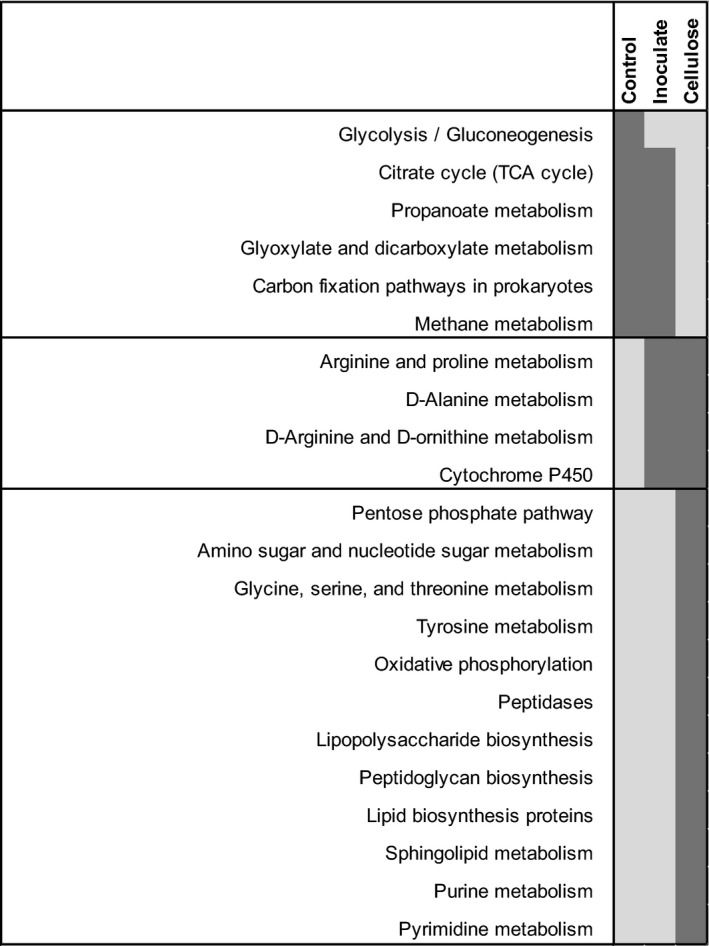
Heatmap indicating significant differences in the proportion of metabolic pathways predicted from 16S rRNA for the three soil amendment treatments at elevated temperature. Significance is assessed at α = 0.001, dark shading indicates that the proportion of the predicted pathway is higher. Data S3.

Similar to the results at ambient temperature, there was no effect of soil amendment on plant AGB or BGB or the biomasses of individual seedlings at elevated temperature (Appendix S1: Figs. S2, S3). For most variables, the remnant microbial inoculation addition did not differ from the control. The only exception was that remnant inoculation stimulated cellobiohydrolase activity at elevated temperature (Fig. 1). pH, total phosphorus and SOM were all higher with cellulose addition than in the control, though this effect was only significant for pH with one plant species and SOM with three plant species (Appendix S1: Fig. S4). Additionally, percent soil carbon was higher in cellulose‐amended soils with *A. gerardii* alone than in the control at elevated temperature. Only when there were three plant species, cellulose addition stimulated cellobiohydrolase, β‐glucosidase, and N‐acetylglucosaminidase activities above what we observed in the control (Fig. 1). Phosphatase activity did not change with different soil amendments, and enzyme activities did not differ with cellulose addition when only *A. gerardii* was present. As at ambient temperature, total lipid biomass was not influenced by cellulose addition, but in contrast, the fungal : bacterial lipid ratio also did not differ, indicating that cellulose did not stimulate the growth of fungal lipids at elevated temperature as it had at ambient temperature (Appendix S1: Fig. S5). Lipid‐based community composition was not affected by cellulose addition and none of the indicator lipids that changed with cellulose addition at ambient temperature differed at elevated temperature (Fig. 2). In contrast, the 16S rRNA‐based bacterial community was more responsive to cellulose addition at elevated temperature than the lipid‐based community. Cellulose addition had a pronounced effect on 16S rRNA‐based communities at elevated temperature in both plant treatments (Fig. 3). As at ambient temperature, the relative abundances of *Actinobacteria* and *Firmicutes*, both of which are copiotrophic phyla, and *Chloroflexi* all decreased significantly with cellulose addition (Fig. 3B). However, under elevated temperatures, *Bacteroidetes* (copiotrophic), *Verrucomicrobia* (oligotrophic), and OTUs associated with low‐abundance phyla all increased with cellulose addition and *Gemmatimonadetes* decreased. While there was no effect of plant species on predicted metabolic pathways, there was a strong effect of cellulose addition at elevated temperature (Appendix S1: Fig. S6). Predicted pathways indicating allocation of carbon to biomass, such as pentose phosphate pathway, peptidoglycan, lipid, and lipopolysaccharide biosynthesis were higher in the treatments with cellulose added (Fig. 5). Additionally, pathways indicating possible nitrogen metabolism, such as nucleic acid, amino sugar, and certain amino acid pathways, were also more represented in the cellulose treatment than in the control, which could indicate nitrogen limitation at elevated temperature. Conversely, predicted pathways related to carbon‐substrate metabolism, such as glycolysis, citric acid cycle, propanoate, glyoxylate, and dicarboxylate and methane metabolism were all higher in the control than in the cellulose treatment.

## Discussion

### Effects of plant species at ambient temperature

Integrating above‐ and belowground restoration practices may improve soil‐based ecosystem services, but further testing is required to determine the effects of belowground restoration strategies in the context of both the plant community and future climate scenarios. From an aboveground restoration perspective, increasing plant diversity and improving vegetation structure are viewed as crucial restoration strategies for obtaining desired ecosystem service outcomes, such as improving pollinator populations, reducing invasive plant encroachment, and improving soil carbon storage (Ruiz‐Jaen and Aide 2005, Eisenhauer et al. 2013, Lange et al. 2015), but this approach is not typically coupled with direct belowground restoration strategies. We acknowledge that prairie restoration efforts typically use a far higher plant seedbank diversity than we used in our experiment. Our work is meant to provide a deconstructed test of how plant community composition directly influences soil microbial communities, and their responses to the other treatments.

Overall, our results suggest that at ambient temperatures, changes in the plant community feed changes in soil microbial communities, but when temperatures increase, plant‐driven differences in microbial communities are reduced. We used seedlings of three later‐successional prairie plants because several studies indicate that early seedling establishment is when *A. gerardii* is most sensitive to competition (Foster 1999, Suding and Goldberg 1999, Gustafson et al. 2004). Plant competition between species, resulting in differences in root biomass, likely explain our observation that predicted microbial pathways for simple sugar, starch and amino sugar metabolisms were higher in soils with only *A. gerardii*. Higher root biomass is associated with greater root exudate production, where exudates generally contain an abundance of the substrates that support the corresponding, more abundant bacterial pathways (simple sugars, starches, and amino acids; Eisenhauer et al. 2017). In addition, production of certain root exudates, such as oxalic acid, can dissociate recalcitrant organic compounds from the soil mineral matrix, increasing carbon availability (Keiluweit et al. 2015). However, greater plant diversity is also associated with more diverse root exudates as well as higher exudate concentrations (Lange et al. 2015, Eisenhauer et al. 2017), which can lead to higher total microbial biomass, as we saw in our three‐plant‐species treatment control.

Plant treatments had few effects on the overall composition of lipid or 16S rRNA‐based communities at ambient temperature, though some selected taxa varied by plant treatment. With the over‐representation of labile substrate metabolism pathways, we expected to see increases in copiotrophic bacterial taxa in the one‐plant‐species treatment, but this was not the case. Since many predicted amino acid pathways were higher in the one‐plant‐species treatment, and percent soil nitrogen did not differ between the plant treatments, this suggests that soil communities in the one‐plant‐species treatment were at least more active, and likely more N‐limited, than in the three‐plant‐species treatment. Nitrogen fertilization is a well‐documented factor influencing the shift from oligotrophic (*K*‐selected) to copiotrophic (*r*‐selected) microbial taxa (e.g., Fierer et al. 2012), and the newly restored soil we used for this experiment had a long history of agricultural fertilization. By discontinuing nitrogen addition, we created a nitrogen limitation effect in both plant treatments, which was stronger with *A. gerardii* alone because of the greater root biomass associated with that species. Competition between *A. gerardii* and copiotrophic taxa for available N, as well as slightly lower pH in the one‐plant‐species treatment, is likely related to the significant increase we observed in the relative abundance of *Acidobacteria*, an oligotrophic phylum. Observations in the *A. gerardii* treatments could also be confounded by size limitations of the pots in the mesocosm experimental design. Larger *A. gerardii* roots may reach the limits of nitrogen present in the pots more quickly than the plant roots in the three‐plant‐species treatment, causing an experimental N‐limitation effect. Further studies conducted in the field and across multiple growing seasons are required to examine this interaction.

*Gammaproteobacteria* were also more abundant in the one‐plant‐species treatments than in the three‐plant‐species treatments. *Gammaproteobacteria* is not one of the six phyla that typically predominate soils (Fierer et al. 2007). We expect that the predominance of *Gammaproteobacteria* in our experiment is because we obtained the newly restored soil from an area with a history of corn/soy rotation. This phylum contains several crop‐plant pathogens within the order *Xanthomonadales* (Kaewnum et al. 2005, Korus et al. 2017), and this order represented the highest proportion of *Gammaproteobacteria* in our soils (2.4% on average). *Xanthomonadales* were more abundant in the one‐plant‐species treatment than in the three‐plant‐species treatment. This suggests that higher‐diversity plant restorations may result in fewer plant pathogens in the soil than lower‐diversity restorations, but more work is necessary to determine if this is a broad trend and the underlying mechanism. If these results hold true at field and landscape scales, then seeding with *A. gerardii* alone could result in a shift in the bacterial community that is adapted for bacterial slow carbon turnover and soil carbon storage as microbial biomass, if no further nitrogen is applied to the system. In our study, when plant species richness increases, this effect is no longer present, though total microbial biomass is marginally higher, reflecting previous work that higher plant diversity results in greater soil microbial biomass (Steinauer et al. 2015). In addition, field‐level studies have shown that prairies planted with high diversity can have greater root biomass as compared to those dominated by C_4_ grasses, so further work is needed to determine whether the same trends in microbial community composition hold true in a field study (Klopf et al. 2017).

### Effects of plant species at elevated temperature

A holistic approach to community restoration is further complicated by the need for creating climate‐ready ecosystems. In our study, increasing the temperature fundamentally changed the competitive dynamics of the plant seedlings. *A. gerardii* had a distinct competitive advantage at higher temperature over the other two plant species, due to greater heat tolerance of this C_4_ grass (e.g., Knapp 1985, Larcher 2003, Mainali et al. 2014). In the three‐plant‐species treatment, *A. gerardii* root biomass was significantly higher than *C. lanceolata* and *E. pallida*. This provides greater surface area for water and nutrient exchange, as well as microbial associations for *A. gerardii*, leading to further competitive advantages. In addition, *A. gerardii* shoot biomass equaled *C. lanceolata* shoot biomass at elevated temperature and overall biomass in the one‐plant‐species treatment was higher than in the three‐plant‐species treatment. Previous work conducted at the BioCON experiment in Minnesota suggests that increasing prairie plant species diversity buffers against soil water loss at elevated temperatures, which relates to higher microbial biomass and greater activity of carbon‐ and nitrogen‐cycling enzymes (Steinauer et al. 2015). We did not observe this effect: soil water content and total microbial biomass were both higher with *A. gerardii* alone than with three plant species at elevated temperature. However, the high diversity plots at the BioCON experiment contain a total of 16 plant species, four of which are C_4_ grasses, including *A. gerardii* (Reich et al. 2001). We hypothesize that the moisture and microbial biomass results observed in both our study and at BioCON are related to higher C_4_ grass root biomass than a specific effect of plant diversity.

As a result of increased plant community competition at elevated temperature, we did not observe any differences in predicted soil bacterial metabolic pathways or specific soil taxa, as were seen at ambient temperature. This is likely because the predominance of *A. gerardii* in both plant treatments led to greater similarities between soil communities, reducing the effects of the less competitive plants. The only difference was there was greater microbial biomass associated with *A. gerardii* alone, likely reflecting the higher biomass and root exudation as we propose drove higher microbial biomass in the three‐plant‐species treatment at ambient temperatures. The fungal : bacterial ratio of lipids was not different between the two plant treatments, so this suggests that *A. gerardii* generally supports the growth of soil microorganisms more when temperatures are higher. While greater microbial biomass could become a mechanism for greater belowground carbon storage, without concurrent shifts in the community toward oligotrophic taxa or higher fungal biomass, the residence time of carbon within microbial biomass is unlikely to change. In summary, *A. gerardii* has a competitive advantage at higher temperatures that reduces the effects of plant species richness on soil communities. Using heat‐tolerant and drought‐adapted grasses in new restorations will increase plant and soil microbial biomass, though there is a danger that it will be difficult to sustain diverse prairies at elevated temperatures, as temperature adapted species will have a competitive advantage and dominate growth.

### Effects of soil amendment at ambient temperature

Our most promising soil treatment for accomplishing the goal of increased carbon storage was cellulose amendment. At ambient temperature, cellulose addition had a large impact on microbial community composition. Fungal lipids (18:1 ω 9c and 18:2 ω 6,9c) were more abundant in cellulose‐amended soils and Gram‐positive bacterial lipids (15:0 iso) decreased, thereby increasing fungal : bacterial ratios. This trend occurred in both the one‐plant‐species and three‐plant‐species treatments, but was more pronounced with *A. gerardii* alone. In natural systems, fungi dominate decomposition of lignin, cellulose, and hemicellulose (de Boer et al. 2005) particularly when phosphorus is limiting (Güsewell and Gessner 2009), as is likely the case in this system, despite the addition of a minimal amount of phosphorus with PBS to all our treatments. Increased carbon sequestration with fungal dominance is a trend that varies depending upon soil organic matter chemistry (Grandy et al. 2009) and traits related to stoichiometry, community composition, and physiology, as well as methodology (reviewed in Strickland and Rousk 2010). However, higher fungal : bacterial ratios have been associated with greater carbon sequestration potential in soils (Jastrow et al. 2007), largely because fungal biomass turnover occurs over the course of several months (Rousk and Bååth 2007), whereas bacterial biomass turnover occurs on the order of a few days (Bååth 1994). In addition, fungal necromass, which contains chitin and ergosterol, is more chemically recalcitrant than bacterial biomass (Zhao et al. 2005). Soils with higher fungal : bacterial ratios are expected to produce necromass that results in longer carbon residence time and greater carbon sequestration (Guggenberger et al. 1999, Strickland and Rousk 2010), though no studies to date have explicitly examined decomposition rates of fungal and bacterial necromass from complex communities (Strickland and Rousk 2010). We only observed an increase in soil organic matter with cellulose addition when *A. gerardii* was present alone, which may be related to the increased saprotrophic or mycorrhizal fungal biomass with cellulose addition. Using PLFA, we were only able to observe increases in general fungal lipids (18:1 ω9c and 18:2 ω6,9c); we did not observe changes in the abundance of 16:1ω5c, which can be produced either by *Glomus* (AMF) or Gram‐negative bacteria (Ngosong et al. 2012). However, 18:1 ω9c is also abundantly made by some AMF genera (Graham et al. 1995). While AMF are not known to use cellulose directly, bacterial byproducts of cellulose degradation may promote fungal growth. As a result, they will form more associations with the more abundant *A. gerardii* root biomass, driving a positive feedback (Anderson et al. 1994).

Within the bacterial community, relative abundances of two copiotrophic bacterial taxa, *Actinobacteria* and *Firmicutes*, decreased with cellulose addition at ambient temperature. This was accompanied by a nonsignificant increase in *Verrucomicrobia*. We did not see an increase in *Acidobacteria*, the other oligotrophic phylum adapted to more acidic soil conditions. Our soil conditions, ranging from pH 5.5 to 7, may not have been optimal for these bacteria to respond to treatment. Taken together, these results suggest that cellulose addition selects against taxa with faster growth rates, favoring some oligotrophic taxa.

In comparison to cellulose addition, we did not observe any significant effects of remnant community inoculation at ambient temperature. The original community used to prepare the inoculate was different from the restored soil used in the experiment, with notably greater abundance of *Verrucomicrobia*, a target bacterial phyla for promoting slow bacterial growth and carbon retention. There could be multiple reasons for our lack of treatment effect. First, the remnant community contained higher relative abundances of *Bacteroidetes* than in the restored soil, and equal abundances of *Betaproteobacteria* and *Actinobacteria*, all of which are adapted to fast growth and labile carbon use. It is possible the remnant soil we used was not different enough from the restored soil to produce an effect. The oligotrophic group, *Acidobacteria*, was equally abundant in both soils, limiting the effectiveness of the remnant inoculate for carbon retention. We envision that an improved approach could use novel soil microbial communities adapted from local remnant sources toward higher abundances of both *Acidobacteria* and *Verrucomicrobia* instead of communities derived directly from a remnant. Second, our extraction procedure may have favored colonization of disturbance‐adapted “weedy” bacterial groups instead of oligotrophs. This could also be because our cellular extraction procedure removed cofactors or rare community members that were required for the establishment of the predominant members of the community. Similarly, buoyant spores and fungal hyphae could also have been removed during extraction. We used a cellular extraction procedure to avoid adding nutrients and soil carbon associated with the remnants, which could have biased our results, but addition of whole soil may be required to achieve an effect. Third, if slower‐growing members of the remnant community were successfully added to the restored soil, they could have been outcompeted by fast‐growing taxa present in the restored community. If this is the case, then a more concentrated remnant inoculate and concurrent addition of a recalcitrant carbon substrate may be required to aid in community establishment. Finally, microbial inoculation may be unnecessary if microbial communities already existing in restored soils can be cultivated by adding a complex carbon source. It was beyond the scope of our study to determine whether these strategies could improve the inoculation procedure, but this should be addressed with future research before this technique is implemented at a field‐scale level.

### Effects of soil amendment at elevated temperature

At ambient temperature, cellulose addition is a promising strategy for increasing in fungal biomass and the relative abundance of *Verrucomicrobia*, regardless of the number of plant species. When only *A. gerardii* is present, cellulose especially stimulates fungal biomass and increases soil organic matter content. In contrast, at elevated temperatures, cellulose addition had no impact on lipid‐based communities, did not stimulate fungal biomass production and did not lead to increases in soil organic matter. Soil fungal responses to climate change can vary across ecosystems (i.e., Allison et al. 2010, Hayden et al. 2012) and be unresponsive in native tallgrass prairies (Jumpponen and Jones 2014). Warming stress can lead to declines in fungal decomposition activity (Allison et al. 2011) and it is possible that warming stress or related nutrient stress prevented fungi from responding positively to cellulose addition under warming as they did under ambient conditions.

Juxtaposed against the lack of effect on fungi, cellulose addition during warming drove even greater differences in bacterial community structure and function. As at ambient temperature, *Actinobacteria* and *Firmicutes* decreased in relative abundance with cellulose addition and *Verrucomicrobia* increased in relative abundance. However, relative abundances of *Bacteroidetes,* a copiotrophic phylum, also increased. It is possible that certain taxa within *Bacteroidetes* are adapted to faster growth at higher temperatures, but previous work suggests that *Bacteroidetes* are selected against by warming in a variety of ecosystems (Luo et al. 2014). Alternatively, reduced competition with fungi for resources, such as nitrogen, at elevated temperatures may have created a new ecological niche, which was filled by fast‐growing *Bacteroidetes* taxa. Predicted metabolic pathway data suggests that the cellulose‐amended community was nitrogen limited, resulting in high representation of amino acid metabolism pathways, as well as stimulated N‐acetylglucosaminidase enzyme activity. Cellulose addition also stimulated pathways favoring allocation of resources toward carbon storage as bacterial biomass, though fungal : bacterial ratios remained the same in all soil amendments. These results are consistent with previous work that indicating that warming stimulates cellulose degradation and CO_2_ production in temperate grasslands (Luo et al. 2014). Because we did not measure bacterial turnover rates, and because DNA and lipids turnover at different rates in the soil, it is possible that the shift we observed in bacterial growth metabolism pathways were not observable in lipid biomass data. In summary, soil fungal communities lost responsiveness to cellulose, and fungal biomass production was not stimulated by cellulose at elevated temperature. In contrast, warmed bacterial communities responded in a similar way to cellulose addition as ambient temperature communities, but also harbored greater abundances of *Bacteroidetes*, which are adapted for fast growth at higher temperatures.

## Conclusions

Integrating aboveground and belowground restoration practices to produce climate‐ready ecosystems is a major challenge faced by land managers, farmers, and restoration scientists. Our results demonstrate that coupling addition of cellulose to soils during restoration plus use of heat and drought‐tolerant grasses can lead to selection of microbial communities that have higher fungal biomass and slower‐growing bacterial taxa. As a result, soils may be adapted for greater carbon retention in the long term, if temperatures remain ambient, but further studies are necessary to determine whether this approach is tractable on a field‐level scale and whether continued cellulose addition is required. Adding raw cellulose is also an expensive endeavor at a field‐scale level, but other less expensive options may work just as well, such as mowed plant biomass. Sawdust has been used previously as an inexpensive way to increase soil C in prairie restorations, but it does not increase soil microbial biomass (Baer and Blair 2008) and other effects on soil microbial community composition are not known. If temperatures continue to increase, our results suggest cellulose addition may still be useful, but fungal biomass will not increase with cellulose addition, as fungi are likely outcompeted by faster‐growing bacterial taxa. In addition, heat‐ and drought‐tolerant grasses may outcompete other plant species that are imperative for improving aboveground ecosystem services, such as insect pollination and bird migrations. Further strategies are necessary to promote climate‐ready restoration practices that are well integrated to promote both above‐ and belowground ecosystems, including developing novel soil communities that are adapted to both heat and carbon retention.

## Data Availability

Data available from the NCBI Sequence Read Archive under Accession no. PRJNA454440.
