# Supplementary material for: Soil microbial restoration strategies for promoting climate‐ready prairie ecosystems
Source: Ecol Appl. 2019 Mar 4;29(3):e01858. doi: 10.1002/eap.1858 (PMC9286448; doi:10.1002/eap.1858)
Supplement: Supplementary file 1 [file EAP-29-e01858-s006.pdf]

Supporting Information. Docherty, K. M. and J. L. M. Gutknecht. 2019. Soil microbial restoration strategies for promoting climate-ready prairie ecosystems. *Ecological Applications.*

Appendix S1.

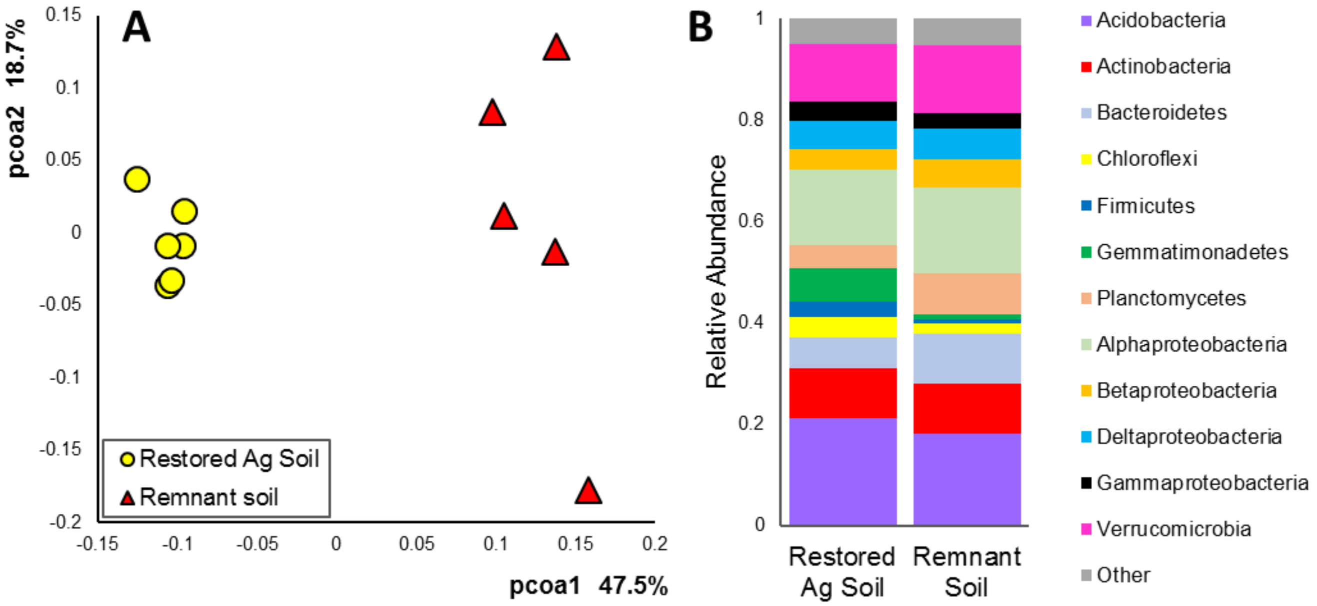

Figure S1. A) Principal coordinates analysis ordination using weighted UniFrac distances indicates that soil bacterial communities in the remnant soil used to prepare the inoculation treatment (red triangles) differed significantly from bacterial communities in the agricultural soil undergoing restoration (yellow circles) used for the experiment (permanova, $p = 0.004$). B) Relative abundances of phyla representing > 5% of the total community for the two soil types.

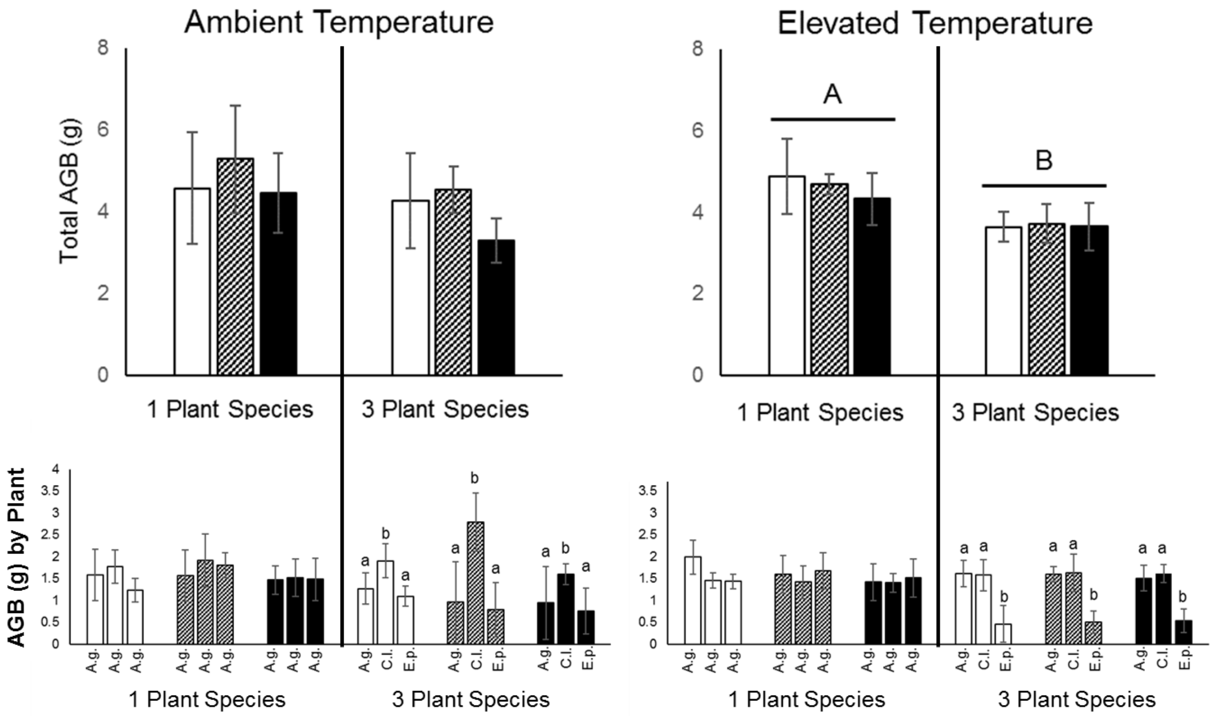

Figure S2. Top panel: Total aboveground biomass (AGB) of all plants in each experimental treatment. There is no difference in total AGB between the 1-plant species and 3-plant species treatments at ambient temperature (left, $p = 0.115$). At elevated temperature (right), total AGB is higher in the 1-plant species treatment than in the 3-plant species treatment ($p = 0.0004$). Plant effects were independent of the soil treatments: control (white), remnant inoculate (lines) and cellulose (black), which did not significantly affect AGB. Bottom panel: AGB of each of the individual seedlings, where A.g. is *Andropogon gerardii,* C.l. is *Coreopsis lanceolata* and E.p. is *Echinacea pallida.* Biomasses of individual seedlings only differed in the 3-plant species treatments. At ambient temperature (left), *C. lanceolata* biomass was higher in all three soil treatments than both *A. gerardii* and *E. pallida* biomasses ($p < 0.0003$). At elevated temperature (right), *E. pallida* biomass was lower than both *A. gerardii* and *C. lanceolata* biomasses in all soil treatments ($p < 0.0001$). DataS1.

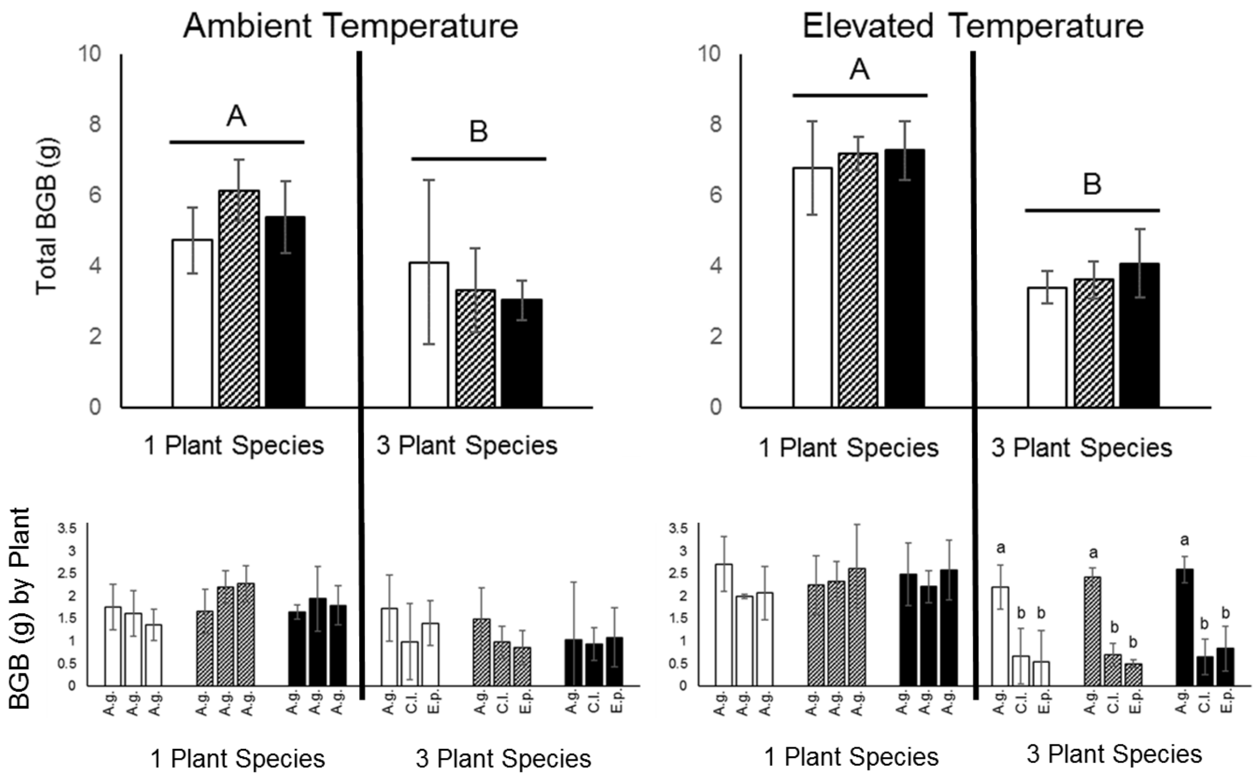

Figure S3. Top Panel: Total belowground biomass (BGB) of all plants in each experimental treatment. At both ambient (left) and elevated (right) temperatures, BGB is higher in the 1-plant species treatment than in the 3-plant species treatment, independent of the soil treatments: control (white), remnant inoculate (lines) and cellulose (black). Bottom panel: Bottom panel: BGB of each of the individual seedlings, where A.g. is *Andropogon gerardii,* C.l. is *Coreopsis lanceolata* and E.p. is *Echinacea pallida.* Biomasses of individual seedlings only differed in the 3-plant species treatment at elevated temperature (right). *A. gerardii* BGB was higher than the other two species, independent of the soil treatment ($p < 0.0001$). DataS1.

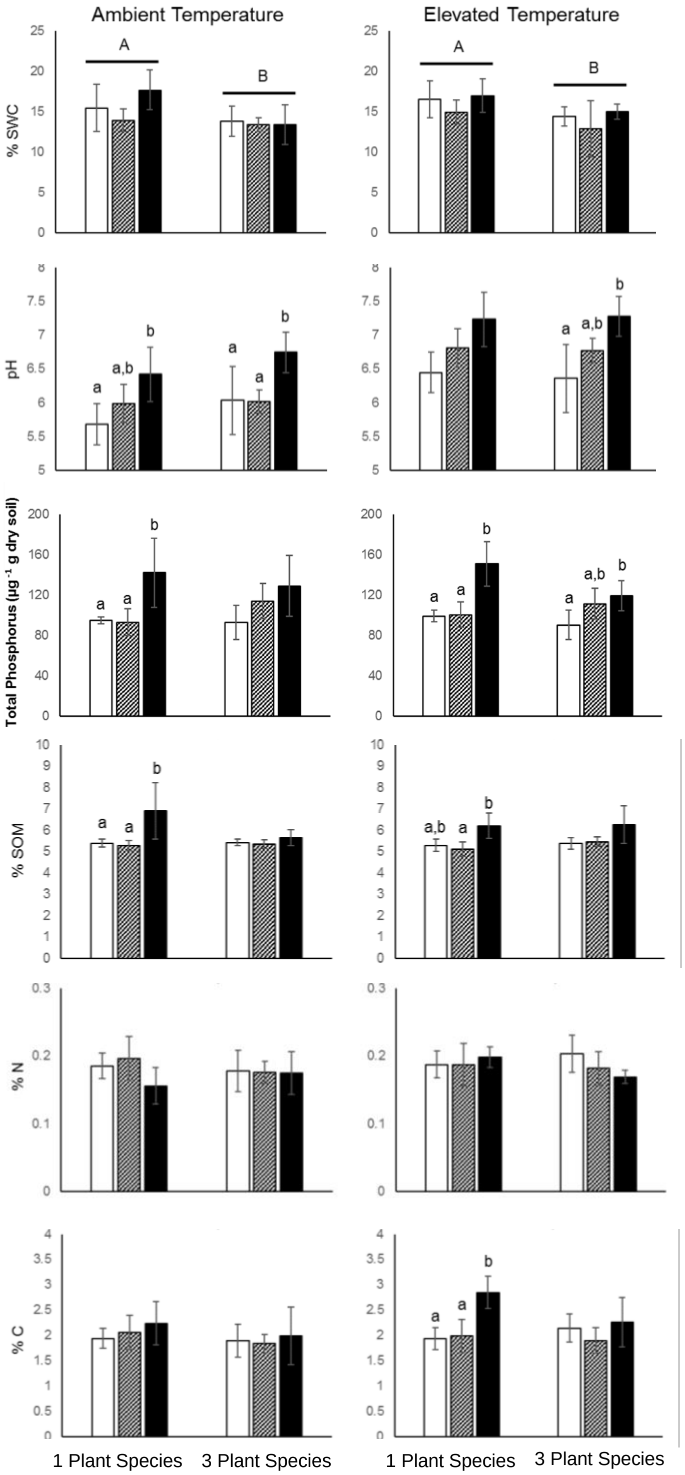

Figure S4. Six soil physiochemical properties for each soil treatment (control is white, remnant inoculate is lined, cellulose is black) for 1-plant and 3-plant species treatment at ambient (left) and elevated (right) temperatures. Average values ± 95% confidence intervals are expressed for all measurements and significance is assessed at $\alpha = 0.05$. From top to bottom, percent soil water content (%SWC), differs by plant treatments, where %SWC is higher in the 1-plant species treatment than in the 3-plant species treatment at both temperatures ($p = 0.0169$). Soil pH is always highest in the cellulose amended treatments ($p < 0.009$), independent of other factors. Total phosphorus ($\mu g\ g^{-1}$ dry soil) is always highest in the cellulose treatment ($p < 0.026$), though this is not significant at ambient temperature with 3-plant species. Percent soil organic matter content (%SOM) is higher in the cellulose treatment at both temperatures, only when 1-plant species is present ($p < 0.023$). Percent total soil nitrogen (%N) does not differ with any treatment combination. Percent total soil carbon (%C) is significantly higher in the cellulose amended treatment at elevated temperature when 1 plant species is present ($p = 0.028$). Data S1.

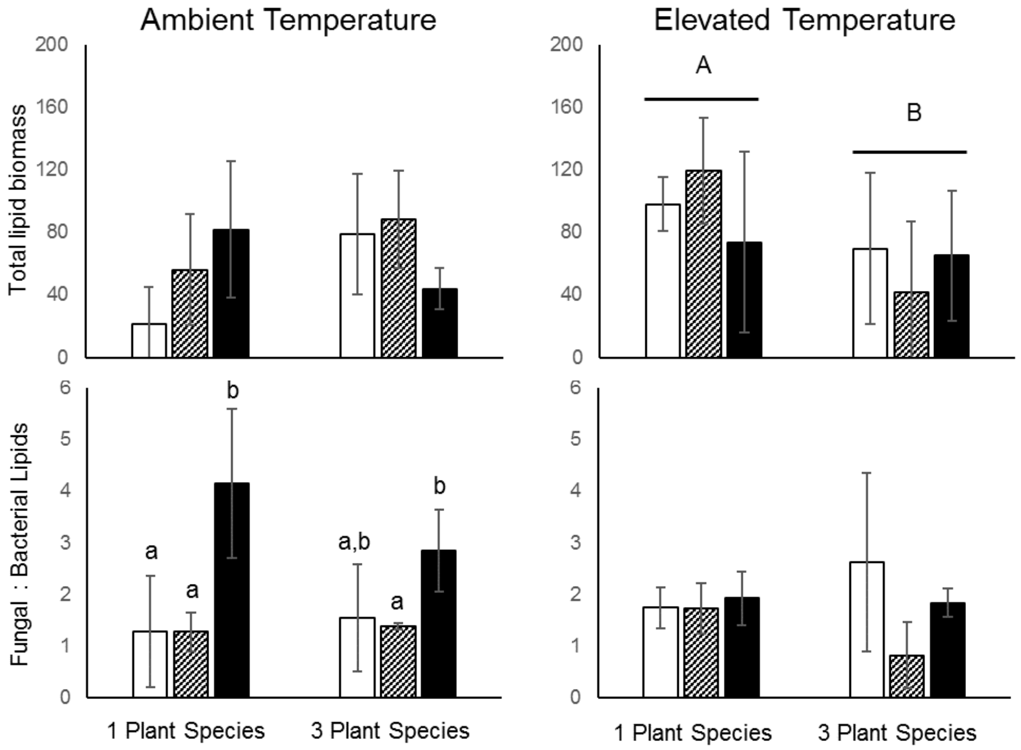

Figure S5. Total microbial lipid biomass (top), expressed as the average sum of all lipids identified ± 95% confidence intervals and fungal : bacterial lipid ratios (bottom). Soil treatments are control (white), remnant inoculate (lined) and cellulose (black), by plant species treatments and ambient (left) and elevated (right) temperatures. Significance is assessed at $\alpha = 0.05$. At elevated temperature only, total microbial lipid biomass is higher in the 1-plant species treatment than in the 3-plant species treatment, independent of soil amendments ($p = 0.045$). At ambient temperature only, cellulose activity is associated with a significant increase in the fungal : bacterial ratio ($p = 0.0008$). DataS2.

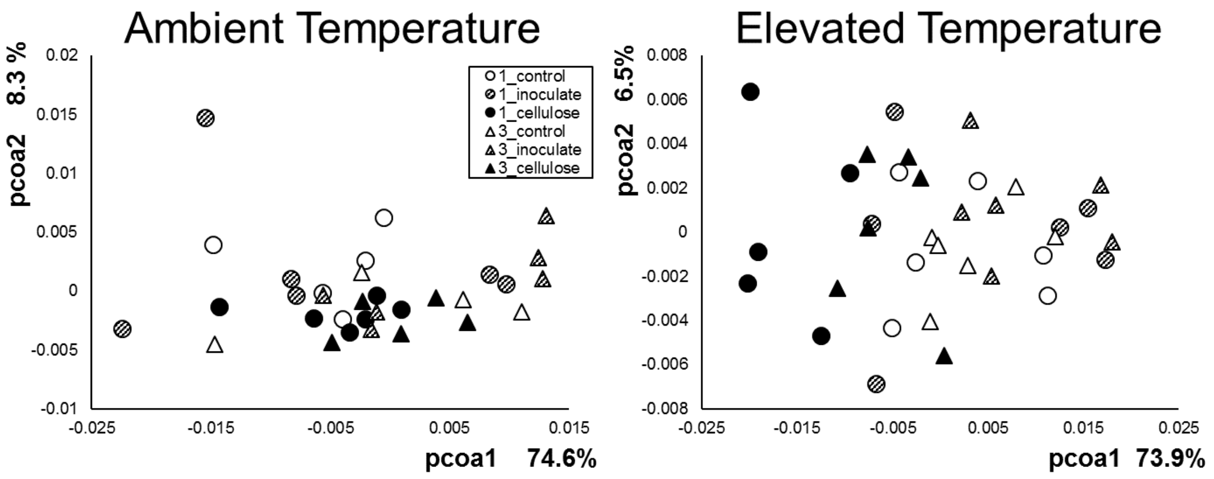

Figure S6. Principal coordinates analysis ordinations based on Bray-Curtis distances between community metabolic pathways predicted from 16S rRNA data for all crossed plant x soil amendment treatments at ambient (left) and elevated (right) temperatures. Significance is assessed at $\alpha = 0.05$. At ambient temperature, there is a significant effect of plant treatment ($p = 0.007$) on predicted metabolic pathways, independent of any of the soil treatments, which did not differ ($p = 0.481$). At elevated temperature, the plant treatment has a marginal effect ($p = 0.069$), but soil amendment is associated with significant differences in community metabolism ($p = 0.002$). Amendment effect is driven by the cellulose treatment, which differs from both the control and remnant inoculate for both plant treatments ($p < 0.009$).

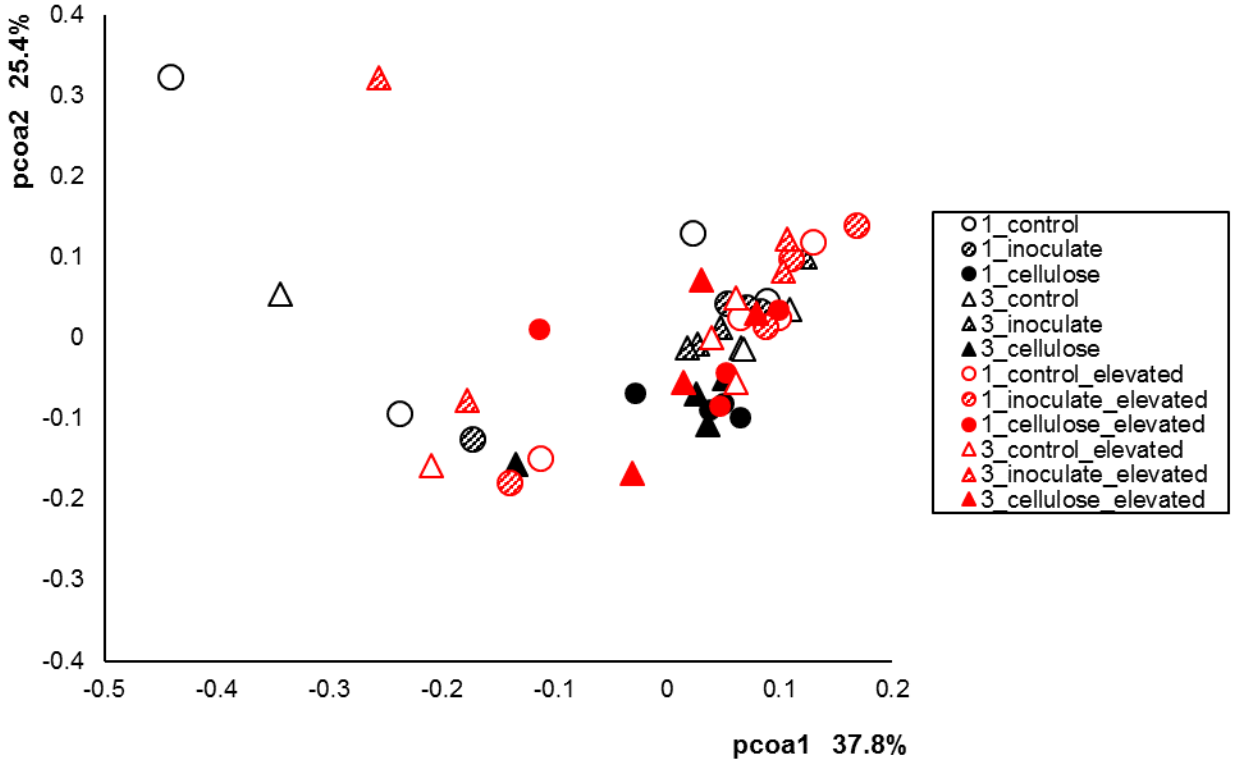

Figure S7. Principal coordinates analysis ordination for lipid-based microbial communities for all treatments. Overall, there is a significant effect of soil amendment on lipid-based communities, which is driven by the effects of cellulose (permanova $p = 0.032$). There is no effect of elevated temperature (red, permanova $p = 0.311$) or plant treatment (shapes, $p = 0.997$) and there are no interactions between treatments ($p > 0.1$ for all interactions). Data S2.

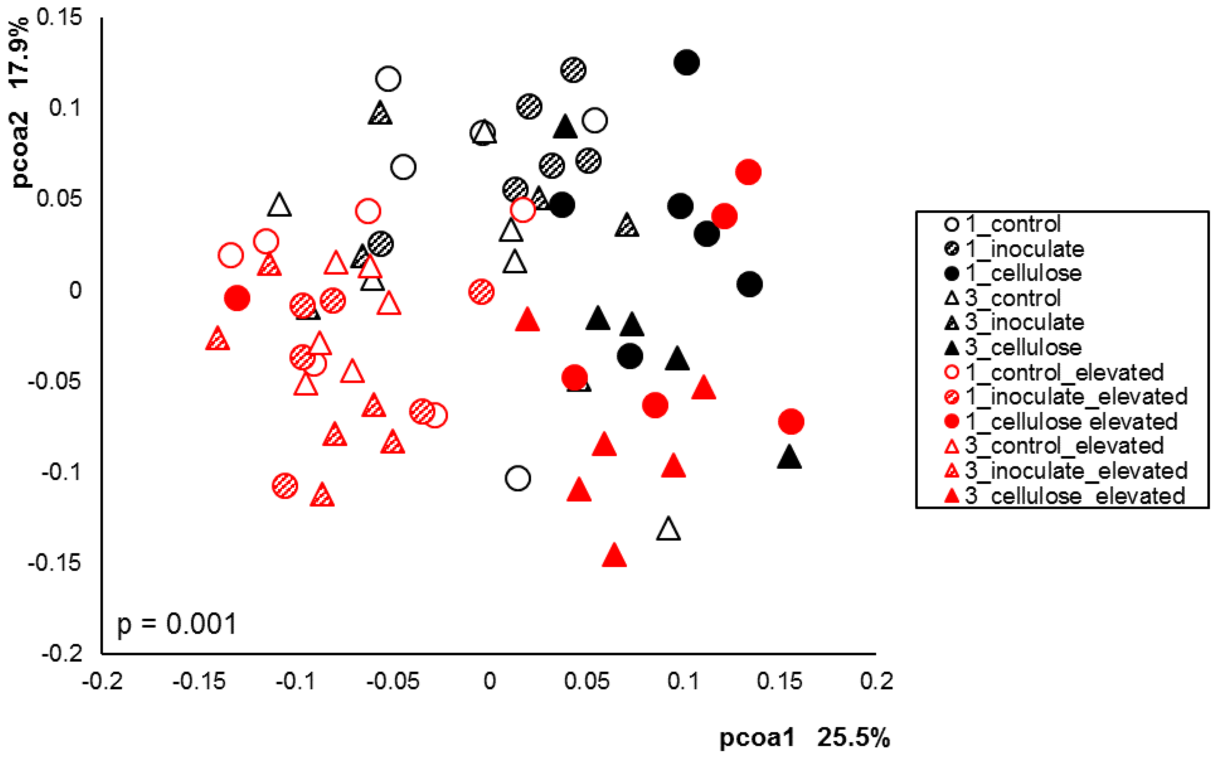

Figure S8. Principal coordinates analysis ordination based on weighted UniFrac distances between bacterial communities analyzed using 16S rRNA amplicons for all treatments. There is a significant effect of temperature (red, permanova, $p = 0.001$).
