# Supplementary material for: Soil microbial restoration strategies for promoting climate‐ready prairie ecosystems
Source: Ecol Appl. 2019 Mar 4;29(3):e01858. doi: 10.1002/eap.1858 (PMC9286448; doi:10.1002/eap.1858)
Supplement: Supplementary file 5 [file EAP-29-e01858-s007.pdf]

Docherty, K. M. and J. L. M. Gutknecht. 2019. Soil microbial restoration strategies for promoting climate-ready prairie ecosystems. *Ecological Applications.*

Data S1

All Univarate Data, including all soil physiochemical properties, extracellular enzyme activities, lipid biomass, fungal:lipid ratio, plant biomass data

---

Authors

Kathryn M. Docherty
Western Michigan University
Department of Biological Sciences
1903 West Michigan Ave., Mailstop 5410, Kalamazoo, MI 49008
kathryn.docherty@wmich.edu

Jessica L.M. Gutknecht
University of Minnesota, Twin Cities
Department of Soil, Water and Climate
439 Borlaug Hall, 1991 Upper Buford Circle, St. Paul, MN 55108
jgut@wmich.edu

File list (file found within DataS1.zip)

`DataS1.csv`

Description

`DataS1.csv` contains all univariate data associated with the manuscript. This includes the pot ID number, the plant species treatment classification (1 or 3), the room temperature classification (ambient or elevated), the soil amendment treatment (control, inoculate or cellulose), soil water content (%SWC), pH, soil organic matter content (%SOM), total phosphorus (ug/g dry soil), total nitrogen (%), total carbon (%) total lipid biomass as measured by PLFA, the ratio of fungal-to-bacterial lipids as measured by PLFA, B-glucosidase enzyme activity (nmol/g ODE soil/h), cellobiohydrolase activity (nmol/g ODE soil/h), N-acetylglucosaminidase enzyme activity (nmol/g ODE soil/h), phosphatase enzyme activity (nmol/g ODE soil/h), total plant aboveground biomass (g AGB), total plant belowground biomass (g BGB). The file also contains the plant shoot

and root masses (in g) for each seedling within each of the one or three species plant treatments. In the case of the 1-species plant treatments, each *A. gerardii* seedling is labeled 1, 2 or 3. In the case of the 3-species plan treatments, the *A. gerardii* seedling is default as seedling 1.
