# Supplementary material for: Soil microbial restoration strategies for promoting climate‐ready prairie ecosystems
Source: Ecol Appl. 2019 Mar 4;29(3):e01858. doi: 10.1002/eap.1858 (PMC9286448; doi:10.1002/eap.1858)
Supplement: Supplementary file 6 [file EAP-29-e01858-s002.pdf]

Docherty, K. M. and J. L. M. Gutknecht. 2019. Soil microbial restoration strategies for promoting climate-ready prairie ecosystems. ***Ecological Applications.***

Data S2

Proportion Relative Abundance of All Identified Lipids

Authors

Kathryn M. Docherty
Western Michigan University
Department of Biological Sciences
1903 West Michigan Ave., Mailstop 5410, Kalamazoo, MI 49008
kathryn.docherty@wmich.edu

Jessica L.M. Gutknecht
University of Minnesota, Twin Cities
Department of Soil, Water and Climate
439 Borlaug Hall, 1991 Upper Buford Circle, St. Paul, MN 55108
jgut@wmich.edu

File list (file found within DataS2.zip)

`DataS2.csv`

Description

`DataS2.csv` contains the relative abundances of all lipids identified using PLFA for this manuscript. The file includes columns for: pot ID number, the plant species treatment classification (1 or 3), the room temperature classification (ambient or elevated), the soil amendment treatment (control, inoculate or cellulose) and the relative abundances of lipids 12:0_RA, 14:0_RA, 15:0 anteiso_RA,15:0 iso_RA 16:0_RA, 16:0 10 me_RA, 16:1 w5c_RA, 16:1 w7c_RA,16:1 w9c_RA, 17:0 anteiso_RA, 17:0 cyclo_RA, 17:0 iso_RA, 18:0_RA, 18:1 w9c_RA, 18:1 w9t_RA, 18:2 w6,9c_RA, 19:0_RA, 19:0 cyclo_RA.
