# Supplementary material for: Soil microbial restoration strategies for promoting climate‐ready prairie ecosystems
Source: Ecol Appl. 2019 Mar 4;29(3):e01858. doi: 10.1002/eap.1858 (PMC9286448; doi:10.1002/eap.1858)
Supplement: Supplementary file 7 [file EAP-29-e01858-s001.pdf]

Docherty, K. M. and J. L. M. Gutknecht. 2019. Soil microbial restoration strategies for promoting climate-ready prairie ecosystems. *Ecological Applications.*

Data S3

All Metabolism data predicted from 16S rRNA sequence data using Picrust

---

Authors

Kathryn M. Docherty
Western Michigan University
Department of Biological Sciences
1903 West Michigan Ave., Mailstop 5410, Kalamazoo, MI 49008
kathryn.docherty@wmich.edu

Jessica L.M. Gutknecht
University of Minnesota, Twin Cities
Department of Soil, Water and Climate
439 Borlaug Hall, 1991 Upper Buford Circle, St. Paul, MN 55108
jgut@wmich.edu

File list (file found within DataS3.zip)

`DataS3.csv`

Description

`DataS3.csv` contains the relative abundances of all metabolic (KEGG1 = Metabolism) pathways predicted from the 16S rRNA amplicon data using the Phylogenetic Investigation of Communities by Reconstruction of Unobserved States tool (PICRUSt), including those used in this manuscript. The file includes columns for: pot ID number, the plant species treatment classification (1 or 3), the room temperature classification (ambient or elevated), the soil amendment treatment (control, inoculate or cellulose). Pathways are identified by KEGG 1-3 classification in rows 3-5. There are 14 columns for pathways associated with Amino Acid Metabolism (F-S); 15 columns for pathways associated with Carbohydrate metabolism (T-AH), 9 columns for pathways associated with Energy Metabolism (AI-AQ); 3 columns for pathways associated with Enzyme Families (AR-AT); 14 columns associated with pathways for Glycan Biosynthesis (AU-BH); 17 columns associated with pathways for Lipid Biosynthesis (BI-BY); 9 columns

associated with pathways for Other Amino Acid Metabolism (BZ-CH); 2 columns associated with pathways for Nucleotide Metabolism (CI-CJ); 20 columns associated with pathways for Xenobiotics Biodegradation and Metabolism (CK-DD). Original sequence files and metadata are uploaded to the NCBI Sequence Read Archive under accession number PRJNA454440.
